# Occupational illnesses in the 2009 Zambian labour force survey

**DOI:** 10.1186/1756-0500-3-272

**Published:** 2010-10-27

**Authors:** Adamson S Muula, Emmanuel Rudatsikira, Seter Siziya

**Affiliations:** 1 Department of Public Health, Division of Community Health, College of Medicine, University of Malawi, Blantyre, Malawi; 2 Division of Epidemiology and Biostatistics, Graduate School of Public Health, San Diego State University, San Diego, California, USA; 3 Department of Community Medicine, School of Medicine, University of Zambia, Lusaka, Zambia

## Abstract

**Background:**

Occupational health has received limited research attention in the Southern African Development Community (SADC). Much of the published data in this region come from South Africa and little has been reported north of the Limpopo. The present study was conducted to estimate the burden of occupational illnesses in Zambia and assess factors associated with their occurrence.

**Methods:**

Data were obtained from the Zambian Labour Force Survey of 2009. Frequencies were used to estimate the prevalence of occupational diseases. Logistic regression analyses were conducted to determine the associations between demographic, social and economic factors and reported illness resulting from occupational exposures. Odds ratios (OR) from bivariate analyses and adjusted odds ratios (AOR) from the multivariate analysis together with their 95% Confidence Intervals (CI) are reported.

**Results:**

Data on 59,118 persons aged 18 years or older were available for analysis, of which 29805 (50.4%) were males. The proportions of the sample that reported to have suffered from an occupational illness were 12.7% among males and 10.4% among females (p < 0.001). Overall the proportions of respondents who reported suffering from fatigue, fever and chest infections were 38.8%, 21.7% and 17.1%, respectively. About two thirds (69.7%) of the study participants had stayed away from work due to the illness suffered at work; there was no sex differences (p = 0.216). Older age, being male, lower education level, married/cohabiting or once married (separated/divorced/widowed), and paid employee or employer/self employed were positively associated with having suffered from illness.

**Conclusions:**

The findings from this study call for urgent effort for specific measures to prevent and mitigate the effects of occupational injuries. These interventions may include: public health campaigns, enforcement or change in work policies and regulations. Special attention may have to be made towards those who were more likely to suffer from occupational illnesses.

## Background

Research on occupational illnesses and the pursuit for improved occupational health have largely been reported from high and middle income nations. Data from low income nations are often unavailable and when they do, are incomplete, unreliable or generally describe poor occupational health situations among workers.

There has been growing literature on occupational illnesses in health care workers and agriculture workforce [1-7]. Health workers are exposed to infections or diseases such as tuberculosis, Hepatitis B, human immunodeficiency virus and acquired immunodeficiency syndrome. Meanwhile workers in certain types of agriculture suffer from ill-health resulting from exposure to animals, micro-organisms, plant material dust or chemicals. This may be important in developing nations like Zambia where the majority of the population are in the agricultural sector.

Much of the data on occupational health and safety from the Southern African Development Community (SADC) are from South Africa. The SADC comprises the following countries: Angola, Botswana, Democratic Republic of Congo, Lesotho, Madagascar, Malawi, Mauritius, Mozambique, Namibia, Seychelles, South Africa, Swaziland, Tanzania, Zambia and Zimbabwe. There is paucity of data from the rest of the region, especially north of the Limpopo. Hence the negative impact of poor work conditions is unappreciated and the scientific basis for interventions and policy formulation is to a great extent absent. Loewenson [8], however, has argued that "While the share of world trade to the world's poorest countries has decreased, workers in these countries increasingly find themselves in insecure, poor quality jobs, sometimes involving technologies which are obsolete or banned in industrialized countries". Examples of obsolete technologies include unshielded dangerous machinery and hazardous substances known to cause increase in occupational diseases and accidents [9]. Loewenson [8] further argued that "The occupational illness which results is generally less visible and not adequately recognized as a problem in low income countries." The present study was carried out to assess the burden of occupational illnesses and associated factors in the Zambian workforce.

## Methods

We obtained data from the Central Statistical Office (CSO) [Zambia] through the Work and Health in Southern Africa (WAHSA) Project. A detail on the methodology that is used in the Zambian labour force survey (LFS) is published elsewhere [10]. However, we briefly describe the methodology below.

### Study design and setting

Cross sectional labour force surveys are conducted from time to time by Central Statistical Office of Zambia. The target population for LFS is persons of age 15 years or older. However, for our study we selected only persons of age 18 years or older and currently in employment (whether paid or not).

### Sample size and sampling

The sample size aims to enroll households and this is designed in such a way as to have adequate power to produce estimates for the entire country, urban and rural areas, and for each province. Zambia has nine provinces which are: Central, Copper Belt, Eastern, Luapula, Lusaka, Northern, North-Western, Southern and Western. The administrative hub of the country is in Lusaka province in which the capital city, Lusaka, is situated. Hence, the major economic sector in Lusaka province is the Service sector. The Copper Belt province as the name implies is the seat of Zambia's copper mining efforts. Fishing is the main occupation in Luapula and Western provinces. Peasantry farming (mainly cultivating maize, cotton and groundnuts) is the major economic activity in the rest of the provinces.

A two stage cluster sampling technique is used to draw sampling units. The primary sampling units are Census Enumeration Areas (CEAs), identified from a sampling frame compiled from the 2000 population and housing census. In the second stage of sampling, households are systematically sampled in each CEA and all persons of age 15 years or older in the household are requested to participate in the survey.

### Questionnaire

The composition of the questionnaire used in LFS varies from survey to survey. In the 2009, the Central Statistical Office incorporated questions on: health outcomes, work sector and conditions, work place facilities, work-related injuries and history of compensation from occupational injuries. The design of the questions and definitions used conform to the requirements set by international bodies such as the International Labour Organization (ILO). Questionnaires were administered in the homes of the survey participants by trained research assistants.

### Data analysis

Analyses were conducted using SPSS version 11.5.0. Frequencies were used to estimate the prevalence of occupational illnesses. The Chi-square test was used to compare proportions. The cut off point for statistical significance was set at the 5% level. Logistic regression analyses (bivariate and multivariate) were conducted to determine the level of association between demographic, social and economic factors and occupational illnesses suffered. We used Deviation as the contrast, and the first category of the explanatory variables as the reference. Odds ratios (OR) from bivariate analysis and adjusted odds ratios (AOR) from a multivariate analysis (backward logistic regression) together with their 95% Confidence Intervals (CI) are reported.

## Results

### Socio-demographic description of the sample

Data on 59,118 study participants of age 18 years or older were available for analysis of which 29,805 (50.4%) were males. Sex was not recorded in 5 participants. The socio-demographic distributions of participants by sex are shown in Table 1. Overall, female participants tended to be younger, and once married (separated, divorced or widowed). More female (61.2%) than male (43.4%) participants had completed no more than 7 years of formal education. While more male (55.7%) than female (38.1%) respondents were self employed, a higher proportion of females (52.5%) were unemployed family workers compared to males (21.0%).

**Table 1:** Socio-demographic characteristics of study participants in the Zambia Labour Survey 2009

Characteristic	Male	Female	Total
	**n (%)**	**n (%)**	**n (%)**

**Age (years)**			

5-9	11654 (18.2)	11656 (17.8)	23310 (18.0)

10-14	11099 (17.3)	10958 (16.7)	22057 (17.0)

15-19	9837 (15.3)	9421 (14.4)	19258 (14.8)

20-24	6435 (10.0)	7585 (11.6)	14020 (10.8)

25-34	10399 (16.2)	11268 (17.2)	21667 (16.7)

35-44	6802 (10.6)	6699 (10.2)	13501 (10.4)

45-54	3715 (5.8)	3729 (5.7)	7444 (5.7)

55+	4178 (6.5)	4330 (6.6)	8508 (6.6)

Total	64119 (100)	65646 (100)	129765 (100)

**Marital status**			

Never married	24364 (50.8)	19143 (38.7)	43507 (44.6)

Married	21501 (44.8)	22459 (45.4)	43960 (45.1)

Separated	476 (1.0)	1058 (2.1)	1534 (1.6)

Divorced	752 (1.6)	2369 (4.8)	3121 (3.2)

Widowed	821 (1.7)	4356 (8.8)	5177 (5.3)

Cohabiting	70 (0.1)	102 (0.2)	172 (0.2)

Total	47984 (100)	49487 (100)	97471 (100)

**Completed years in formal school**			

0	348 (0.7)	373 (0.7)	721 (0.7)

1-3	10331 (19.5)	11828 (23.1)	22159 (21.3)

4-7	20239 (38.2)	22179 (43.3)	42418 (40.7)

8-9	10312 (19.5)	9060 (17.7)	19372 (18.6)

10-12	9820 (18.6)	6541 (12.8)	16361 (15.7)

13+	1887 (3.6)	1246 (2.4)	3133 (3.0)

Total	52937 (100)	51227 (100)	104164 (100)

**Province**			

Central	6147 (9.6)	6165 (9.4)	12312 (9.5)

Copperbelt	10349 (16.1)	10397 (15.8)	20746 (16.0)

Eastern	7978 (12.4)	8085 (12.3)	16063 (12.4)

Luapula	5937 (9.3)	5970 (9.1)	11907 (9.2)

Lusaka	7162 (11.2)	7260 (11.1)	14422 (11.1)

Northern	8080 (12.6)	8208 (12.5)	16288 (12.6)

North-Western	4378 (6.8)	4407 (6.7)	8785 (6.8)

Southern	8485 (13.2)	8830 (13.5)	17315 (13.3)

Western	5603 (8.7)	6324 (9.6)	11927 (9.2)

Total	64119 (100)	65646 (100)	129765 (100)

Current* employment status			

Self employed	17050 (42.3)	11565 (29.7)	28615 (36.1)

Employed	126 (0.3)	67 (0.2)	193 (0.2)

Paid employee	7641 (18.9)	3253 (8.4)	10894 (13.8)

Unpaid family worker	15406 (38.2)	23896 (61.5)	39302 (49.6)

Other	116 (0.3)	99 (0.3)	215 (0.3)

Total	40339 (100)	38880 (100)	79219 (100)

**Current employer**			

Central government	1549 (3.8)	924 (2.4)	2473 (3.1)

Local government	217 (0.5)	126 (0.3)	343 (0.4)

Parastatal/State owned firm	352 (0.9)	95 (0.2)	447 (0.6)

Private	7382 (18.3)	3529 (9.1)	10911 (13.8)

NGO** or church	251 (0.6)	153 (0.4)	404 (0.5)

International organization	49 (0.1)	24 (0.1)	73 (0.1)

Household	30542 (75.7)	34032 (87.5)	64574 (81.5)

Total	40342 (100)	38883 (100)	79225 (100)

**Current number of employees at place of work**			

5+	11907 (29.6)	8128 (21.0)	20035 (25.4)

Total	40162	38646	78808

**Current employment activity**			

In paid employment/business	18489 (29.1)	10884 (16.7)	29373 (22.8)

In paid employment but temporarily not working due to illness, leave, industrial dispute or on study	235 (0.4)	151 (0.2)	386 (0.3)

Working without pay	11942 (18.8)	16991 (26.1)	28933 (22.5)

Not working but looking for work/business	1533 (2.4)	1118 (1.7)	2651 (2.1)

Not working and not looking for work but available for work/business	4647 (7.3)	5300 (8.1)	9947 (7.7)

Housewife/Homemaker	244 (0.4)	4580 (7.0)	4824 (3.7)

Retired	169 (0.3)	53 (0.1)	222 (0.2)

In school (full time student)	16479 (25.9)	15448 (23.7)	31927 (24.8)

Too old to work	654 (1.0)	1113 (1.7)	1767 (1.4)

Too young to work	7361 (11.6)	7346 (11.3)	14707 (11.4)

Not working, not looking for work and not available for work for other reasons	1878 (3.0)	2184 (3.4)	4062 (3.2)

Total	63631 (100)	65168 (100)	128799 (100)

* status in the previous 7 days to the survey

** Non Governmental Organization

Note: Numbers not adding up due to missing information

### Illnesses suffered at workplace

The proportions of males and females who reported to have suffered from any illness known or suspected to result from work in the past 12 months prior to the survey were 12.7% and 10.4%, respectively. Overall the proportions of respondents who reported suffering from fatigue, fever and chest infections were 38.8%, 21.7% and 17.1%, respectively. About two thirds (69.7%) of the study participants had stayed away from work due to the illness suffered at work; there was no sex differences (p = 0.216), see Table 2.

**Table 2:** Serious illness suffered at workplace in past 12 months prior to the survey.

	Male	Female	Total
**Characteristic**	**n (%)**	**n (%)**	**n (%)**

**Suffered from any illnesses due to work in past 12 months**			

Yes	3660 (12.0)	3027 (9.6)	6687 (10.8)

Total	30558	31525	62083

**Most serious illness suffered from due to work in past 12 months**			

Skin problems	151 (4.1)	110 (3.6)	261 (3.9)

Respiratory problems	268 (7.3)	142 (4.7)	410 (6.1)

Allergies	41 (1.1)	43 (1.4)	84 (1.3)

Diarrhoea	99 (2.7)	139 (4.6)	238 (3.6)

Fatigue	1360 (37.2)	1235 (40.8)	2595 (38.8)

Chest infections	691 (18.9)	428 (14.1)	1119 (16.7)

Fever	738 (20.2)	731 (24.2)	1469 (22.0)

Other	312 (8.5)	198 (6.5)	510 (7.6)

Total	3660 (100)	3026 (100)	6686 (100)

**Stayed away from work due to above Illness**			

Yes	2503 (68.4)	2133 (70.5)	4636 (69.4)

Total	3659	3025	6684

**Days away from work due to above Illness**			

<7	1103 (44.4)	963 (45.6)	2066 (45.0)

7-13	735 (29.6)	604 (28.6)	1339 (29.1)

14-20	322 (13.0)	250 (11.8)	572 (12.4)

21+	325 (13.1)	293 (13.9)	618 (13.4)

Total	2485 (100)	2110 (100)	4595 (100)

**Received compensation from work as a result of the above illness**			

Yes	202 (5.6)	63 (2.1)	265 (4.0)

Total	3635	3011	6646

Note: Numbers not adding up due to missing information.

Table 3 shows the proportions of serious illnesses suffered in relation to work conditions. Fatigue was the most common illness among persons exposed to vibrations (31.7%), breathing in smoke, fumes, powder or dust (40.1%), pesticide (37.6%), skin contact with chemicals (38.4%), handling infectious materials or waste (26.0%), and lifting heavy objects (39.5%). Chest infections were common among persons exposed to temperatures causing perspiration (26.8%), breathing in vapours from other chemicals such solvents and thinners (27.0%), noise (24.2%), and radiation (21.8%). Fever was most common among persons exposed to low temperatures (26.8%).

**Table 3:** Demographic, social and economic factors associated with serious illnesses

	Suffered serious illness		Bivariate	Multivariate
**Factor**	**Total**	**n (%)**	**OR (95%cCI)**	**AOR (95%CI)**

**Age (years)**				

<15	45385	69 (0.2)	1	1

15-19	19262	221 (1.1)	0.55 (0.49, 0.62)	0.69 (0.60, 0.81)

20-24	14023	508 (3.6)	1.78 (1.63, 1.95)	1.69 (1.51, 1.89)

25-34	21669	1458 (6.7)	3.41 (3.18, 3.67)	1.95 (1.76, 2.17)

35+	29459	2382 (8.1)	4.16 (3.89, 4.45)	2.03 (1.83, 2.26)

**Sex**				

Female	65646	2131 (3.2)	1	1

Male	64119	2507 (3.9)	1.01 (1.07, 1.13)	1.11 (1.07, 1.16)

**Completed years in school**				

<3	22983	466 (2.0)	1	1

4-7	42508	1878 (4.4)	1.33 (1.27, 1.40)	1.24 (1.18, 1.31)

8-9	19405	825 (4.3)	1.28 (1.20, 1.36)	0.98 (0.92, 1.05)

10+	19527	647 (3.3)	0.99 (0.92, 1.05)	0.60 (0.56, 0.65)

**Current marital status**				

never married	43512	577 (1.3)	1	1

married/cohabiting	44134	3336 (7.6)	1.87 (1.79, 1.95)	1.24 (1.17, 1.32)

separated/divorced/widowed	9833	700 (7.1)	1.75 (1.65, 1.86)	1.17 (1.08, 1.27)

**Province**				

Western	11928	200 (1.7)	1	1

Copperbelt	20748	661 (3.2)	0.97 (0.90, 1.04)	1.19 (1.09, 1.30)

Eastern	16065	295 (1.8)	0.55 (0.49, 0.61)	0.47 (0.41, 0.53)

Luapula	11907	1071 (9.0)	2.90 (2.72, 3.09)	2.77 (2.56, 2.99)

Lusaka	14446	375 (2.6)	0.78 (0.71, 0.86)	0.88 (0.79, 0.99)

Northern	16288	672 (4.1)	1.26 (1.17, 1.36)	1.13 (1.03, 1.23)

North-Western	8788	597 (6.8)	2.14 (1.97, 2.32)	2.19 (1.99, 2.42)

Southern	17316	431 (2.5)	0.75 (0.68, 0.82)	0.81 (0.73, 0.89)

Central	12312	336 (2.7)	0.82 (0.74, 0.91)	0.81 (0.72, 0.90)

**Current employment status**				

unpaid formal worker	39383	1204 (3.1)	1	1

paid employee	10915	767 (7.0)	1.23 (1.17, 1.30)	1.36 (1.27, 1.46)

employer/self employed	28843	2561 (8.9)	1.59 (1.52, 1.65)	1.03 (0.98, 1.08)

suffered at workplace

Note: Numbers not adding up due to missing information.

### Multivariate analysis of factors associated with illnesses suffered at workplace

All the factors that were significantly associated with having suffered from illness arising from place of work in bivariate analyses remained significant in a multivariate analysis (Table 3). Older age, male, lower education level, married/cohabiting or once married (separated/divorced/widowed), and paid employee or employer/self employed were positively associated with having suffered from illness.

## Discussion

The labour force survey is one of the largest studies conducted in Zambia. Female respondents tended to be less educated, married or were once married and unpaid family workers. More than 10% of workers reported illness they considered to be work related, for which 70% of those affected stayed away from work.

We found that respondents with more education were less likely to suffer from illnesses compared to respondents with little or no education. In a study conducted among Nigerian welders, Sabitu et al (2009) reported that only 20% of those who had no formal education were aware of occupation hazards and safety measures compared to 77.6% among those who has primary education and 85.0% among those who had secondary education [11]. People with education are more knowledgeable to avoid harmful exposures, and as a result may be less likely to fall ill. Educated workers may also be employed in more skilled but less hazardous jobs, and as a result may be less likely to suffer from illnesses.

We also found that workers who were self employed had missed more workdays as a result of work-related illness compared to those employed by others. There are several possible reasons why this may be the case. Firstly, it is possible that self-employed workers are less likely to pay attention to safe work environments as they may be accountable only to themselves. As a consequence, they may be more likely to suffer occupation associated illnesses. Secondly, it is possible the self-employed persons have more opportunity to excuse themselves from work due to illness while it may be harder for those who are employed by others.

Results from our study suggest that males are more likely to suffer from serious illnesses than females. Men may be more likely to work in harsher environment than females, and this may partly explain the observed sex difference in the proportion of serious illnesses suffered.

### Limitations of the study

There are several limitations for this present study. Data were collected through self-reports, and our results may be biased to the extent that the participants mis-reported either intentionally or unintentionally. Since the design of the data collection was cross sectional, it is not possible to assign causation to any of the explanatory variables. We did not have information on the underlying medical conditions [12], and stress [13] to verify the illnesses reported by the respondents as resulting from their workplaces. Information on how the sample size was determined or the participation rate was not obtained from the CSO.

## Conclusion

The prevalence of work-related illness was high in Zambia, and associated with significant levels of absence from work. The data provide good social and socioeconomic grounds to motivate for improvements to working conditions to prevent these occurrences as well as a baseline on which to base statistical targets for improvement. There was geographic variation in the distribution of reported disease, with higher reported prevalence in specific provinces. This information could be useful to the Ministry of Labour to identify areas in specific need of attention, especially in terms of surveillance, enforcement or revision of work policies.

## Competing interests

The authors declare that they have no competing interests.

## Authors' contributions

ASM participated in data collection, interpretation of findings and writing the manuscript. ER participated in the decision of which aspects of the data to analyze, interpretation of data and drafting of the manuscript. SS participated in data collection, analysis and drafting of manuscript. All authors approved the final draft of the manuscript.
